# Training scientists as future industry leaders: teaching translational science from an industry executive’s perspective

**DOI:** 10.15761/JTS.1000214

**Published:** 2018-02-13

**Authors:** Gloria Lee, Jay D Kranzler, Ravichandran Ramasamy, Gabrielle Gold-von Simson

**Affiliations:** 1 T35 NIDDK Honors Trainee, Clinical and Translational Science Institute, New York University School of Medicine, New York, NY, USA; 2 State University of New York at Downstate College of Medicine, Brooklyn, NY, USA; 3 Department of Medicine, New York University School of Medicine, New York, NY, USA; 4 New York University Stern School of Business, New York, NY, USA; 5 Departments of Medicine, Biochemistry and Molecular Pharmacology, co-PI NIDDK R25, New York University School of Medicine, New York, NY, USA; 6 Department of Pediatrics, PI NIDDK R25, New York University School of Medicine, New York, NY, USA; 7 Clinical Translational Science Institute, New York University School of Medicine, New York, NY, USA

**Keywords:** career, drug development, business, education, graduate student, translation

## Abstract

PhDs and post-doctoral biomedical graduates, in greater numbers, are choosing industry based careers. However, most scientists do not have formal training in business strategies and venture creation and may find senior management positions untenable. To fill this training gap, “Biotechnology Industry: Structure and Strategy” was offered at New York University School of Medicine (NYUSOM). The course focuses on the business aspects of translational medicine and research translation and incorporates the practice of business case discussions, mock negotiation, and direct interactions into the didactic. The goal is to teach scientists at an early career stage how to create solutions, whether at the molecular level or via the creation of devices or software, to benefit those with disease. In doing so, young, talented scientists can develop a congruent mindset with biotechnology/industry executives. Our data demonstrates that the course enhances students’ knowledge of the biotechnology industry. In turn, these learned skills may further encourage scientists to seek leadership positions in the field. Implementation of similar courses and educational programs will enhance scientists’ training and inspire them to become innovative leaders in the discovery and development of therapeutics.

## Introduction

Cutting edge, innovative science and technology creates a need for industry-specific training for scientists. Translation of discoveries into healthcare solutions is rife with pervasive, global challenges and an increased need for PhD level researchers and positions. However, most PhDs have little knowledge of industry trends, strategy and structure. Many PhD graduates and post-doctoral researchers are facing a difficult career path ahead, as faculty positions are in short supply as is the funding that is necessary to remain on the tenure track [1]. Hence, many PhDs find themselves in the precarious position of preparing for alternative paths, primarily working within the life sciences industry. In addition, industry jobs are competitive, especially as cutbacks in research and development (R&D) budgets limit the number of open positions. To be successful competing for the limited available jobs, we believe that scientists would benefit from training focused on translating their knowledge of academic R&D into a knowledge base more suitable to the industry setting.

The Clinical and Translational Science Awards (CTSA) initiative of the National Institutes of Health (NIH) National Center for Advancing Translational Science (NCATS) endorses educational and training programs that cultivate leaders in the biomedical research workforce [2,3]. Our institution’s CTSA has demonstrated success with several educational and mentoring programs. One such program is a dual (MD/Master’s) degree program in Clinical Investigation; another is the Drug Development Educational Program [4–6]. However, other time-efficient and cost-effective training programs, particularly those with a focus on the biopharmaceutical industry and acquisition of leadership skills remain scarce. Owing to the importance of economic feasibility and industry trends in determining a project’s success, academic institutions have created MBA/MD, MBA/PhD, and MBA/MS programs. However, these programs can be cost and time prohibitive.

To address this need, in 2015 the NYUSOM implemented a seminar course titled, “Biotechnology Industry: Structure and Strategy” (BISS). BISS is a required course for NYUSOM’s Certificate in Health Innovations and Therapeutics (HIT) program. The goal of the HIT program is to teach essential aspects of drug discovery and development to early career stage scientists and other graduate students with interest in the development of therapeutics [4].

Successful translation of therapeutics from bench to bedside requires lead scientists to think holistically, comprehensively, and strategically, beyond their experience as researchers in the academic setting. Therefore, the course was specifically designed to shift students’ thinking from the focused and deep, yet narrow knowledge base requisite for success in the lab toward more global thinking and decision making that is required to excel in industry. These skills include understanding the many elements that need to be considered in making decisions within the context of industry, including a perspective on the functions within pharmaceutical companies, including R&D, manufacturing, and sales and marketing. Other critical areas for corporate success include an understanding of intellectual property as well as the sources of funding for small development stage companies. Finally, the ultimate success in industry leads to a management position, with demonstrated problem solving and leadership skills differentiating those that are promoted out of the lab to those who remain. In BISS, students are exposed to these functions and have the opportunity to practice these skills through business case discussions and interactions with key players in the biotechnology industry. Here, we describe the BISS course content and student demographics. We also analyze student pre and post course survey results to assess qualitative effectiveness.

## Course description

**Figure 1:**
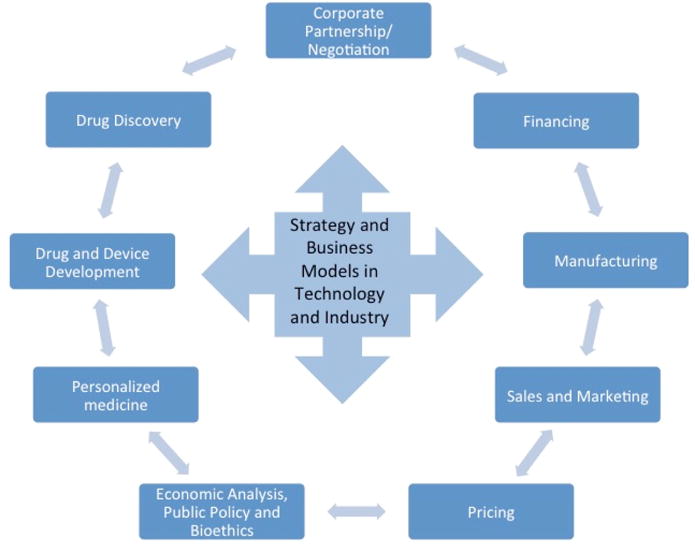
BISS Topics This figure shows the course topics.

The main goal of BISS is to teach students how to identify, analyze, and solve issues from the perspective of a senior manager in a life science enterprise. The course designer/director holds both MD and PhD degrees, founded and served as the CEO of several biotechnology companies, and held leadership positions at major pharmaceutical companies. The course is organized into 12 three-hour sessions. Each session covers a different aspect of the industry. The first two hours of each class are dedicated to a Socratic discussion of pre-assigned business cases led by the course instructor. The topics for discussion include intellectual property, financing, business strategies, sales, pricing, marketing, and manufacturing (Figure 1). The group didactic discussions are meant to help students adopt the perspective of a company CEO or other senior management, rather than the perspective of a lab or research scientist.

The final hour of each session provides a forum for invited speakers to share their experiences and engage in questions and answers with the students. The invited speakers include founders of start-up biotechnology firms, patent attorneys, business consultants, biotechnology investors, and leaders from the NYU Entrepreneurial Institute. The speakers are selected to provide the students with real-life examples of translation of the material covered to application in real situations.

Most notably, two sessions are dedicated to the art of negotiation. The selected discussion case is derived from the instructor’s experience. In this case, a small company is looking to partner with another company to sell a drug, which is developed by a third company for a new indication. Students break up into three groups, each representing a vested company, and are tasked with carrying out a negotiation on that company’s behalf. Doing so effectively requires a combination of analytic ability, and communication skills, as well as industry strategy and tactics. Construction and utilization of financial models are among other key skills essential for upper level managers in the biotechnology industry. In the course, students learn how to build a basic financial model for a biotechnology company and how to utilize such a model as a tool for strategy development and decision-making.

## Student demographics

In its first year as a HIT program required course, BISS enrolled 13 students. 10 (77%) are post-doctoral fellows and 3 (23%) are PhD candidates. 12 (92%) students are affiliated with NYUSOM and 1 (8%) student is enrolled at the NYU Graduate School of Arts and Sciences. 2 students (15%) are registered for the HIT certificate. 7 of the 13 students (54%) are female. All the students are involved in mentored research. Some students are engaged in basic research and study the pathogenesis of disease and others are directly involved in the development of therapeutics.

## Student expectations and learning

### Pre-course analysis

As ascertained by survey data, all the students were interested in careers within the pharmaceutical/device and/or biotechnology sectors. Students considered the course to be greatly relevant to their career goals (pre course survey average: 3.82 (0.40) of 4). In the pre-course survey, one question read, “If you are working/planning to work in the biotechnology industry, in which of the following areas are you most interested?” The answer choices reflected the current departmental structure of most large pharmaceutical companies and included: administration, business development, clinical, compliance, engineering, executive, general management, government affairs, health economics, manufacturing, sales and marketing, medical affairs/services, operations, plant facilities, process development, project management, quality, regulatory, research, and safety. 77% (10) of students selected the research department. All 13 students indicated that their main reason for taking this course was to gain a basic understanding of the biotechnology industry.

### Post-course analysis

In the post course survey, about 50% of students (6) indicated they wanted to either start their own companies or work at the level of a manager/executive. After completion of the course, more students were interested in jobs within business development [pre course survey: 31% (4); post course survey: 54% (7)].

### Knowledge gained

**Figure 2:**
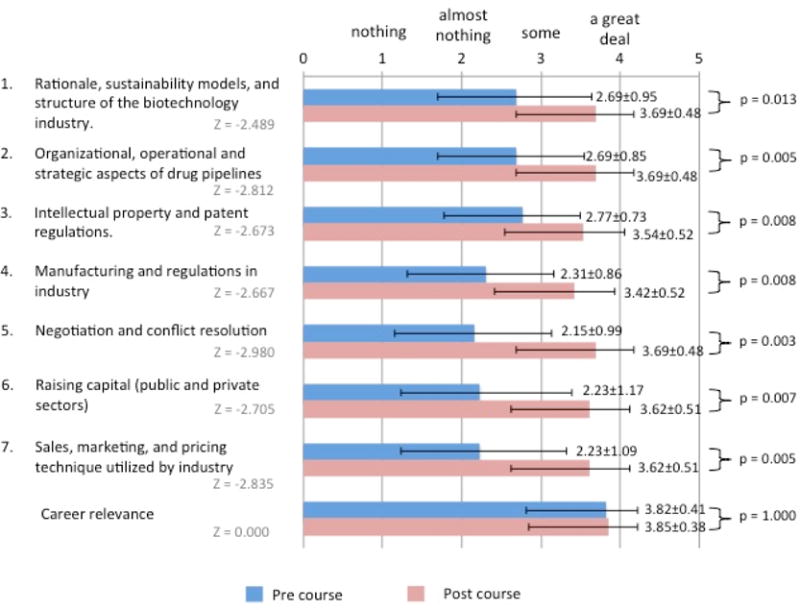
Survey Data from BISS 2017 The results of the pre and post course surveys. 13 students self-rated their knowledge in seven domains and career relevance. Answer choices (scale): (1) nothing, (2) almost nothing, (3) some, (4) a great deal. Means and standard deviations are shown.

Students rated themselves in regard to their knowledge in seven course-relevant domains. Prior to starting the course, knowledge regarding negotiation and conflict resolution ranked lowest (2.15 (0.99) out of 4). After completion of the course, negotiation and conflict resolution was one of the three domains of knowledge where students rated themselves the highest (3.69 (0.48) out of 4). The other two domains were “rationale, sustainability models, and structure of the biotechnology industry” as well as “organizational, operational, and strategic aspects of drug pipeline”. Students rated themselves significantly higher in all domains of knowledge after taking the course. Career relevance was ranked highly in both pre and post-course surveys (Figure 2).

## Lecture ratings and comments

All the case discussions and guest speakers were highly rated (average rating for case discussions: 4.50 (0.65) of 5 and guest speaker lectures: 4.23 (0.89) of 5). The highest rated discussion activity was the mock negotiation session (4.80 (0.42) of 5). The highest rated guest speaker lecture was about negotiation strategies and discussion of how to get involved in the business side of the biotechnology industry (4.82 (0.40) of 5). In the post course survey, students were asked how comfortable they were with applying their research to clinical use; the average rating was 4.08 (0.49) of 5. Other open-ended comments included very positive reviews of the invited experts as well as the course instructor, again citing their experience as well-known leaders in the field.

## Educating scientists beyond the lab

This course augments scientific training by building knowledge about the added complexity of working within the context of a for-profit enterprise. Moreover, the course endorses and reinforces leadership, teamwork and negotiation skills. Students stated they felt comfortable applying their research to clinical use (average rating was 4.08 (0.49) of 5). This may be due to the fact that BISS provided examples of how researchers promoted their discoveries and implemented start up companies. As such, students became more familiar with the path toward commercialization of research. Moreover, BISS brings together students and experts who are actively engaged in building, investing, or leading companies. As a result of these active learning sessions, students are exposed to a different mindset and focus, and they approach the issues like leaders and innovators, rather than *just* scientists.

By inspiring scientists, early in their careers, to think about the economic and business aspects of translational medicine at each stage, courses and programs like BISS and HIT will bridge the translational gap and potentially allow for greater conversion of ideas and discoveries into therapeutics.

## Impact

Educational programs such as BISS enhance early stage researchers’ business skills and complement their scientific skill set to promote translation of scientific discoveries. BISS incorporates case based analysis and discussions with industry experts. The skills learned are traditionally difficult to teach. It is our hope that students will learn not only how to identify issues in delivering discoveries to market but also how to find solutions by bringing together the vital stakeholders. The course has several strengths, but perhaps at the core is the ability to motivate students to think like executives.

To thoroughly examine how BISS influences students’ careers and their ability to carry out translatable research, long term follow up on students’ career trajectories is needed. In summary, we believe that curricula including courses like BISS and programs like HIT are necessary to address the needs of an evolving scientific workforce. Training scientists to think more about the business and economic aspects of discoveries and innovations can lead to increased realization of research product, therapeutics, and healthcare solutions.
